# A rare imaging presentation with multisystemic clinicopathological features of Langerhans cell histiocytosis: Case report and literature review

**DOI:** 10.1097/MD.0000000000034881

**Published:** 2023-09-01

**Authors:** Xiaofen Li, Yulu Wang, Qian Liu, Qingyun Zeng, Huan Fu, Jianlin He, Ingo G.H. Schmidt-Wolf, Amit Sharma, Fengxiang Liao

**Affiliations:** a Department of Medical Imaging, Jiangxi Provincial People’s Hospital, The First Affiliated Hospital of Nanchang Medical College, Nanchang, Jiangxi, China; b Department of Integrated Oncology, Center for Integrated Oncology (CIO), University Hospital Bonn, Germany; c Department of Pathology, Jiangxi Provincial People’s Hospital, The First Affiliated Hospital of Nanchang Medical College, Nanchang, Jiangxi, China; d Department of Nuclear Medicine, Jiangxi Provincial People’s Hospital, The First Affiliated Hospital of Nanchang Medical College, Nanchang, Jiangxi, China; e Hematology department, Jiangxi Provincial People’s Hospital, The First Affiliated Hospital of Nanchang Medical College, Nanchang, Jiangxi, China; f Ping An Haoyi medical imaging center of Nanchang; g Department of Neurosurgery, University Hospital Bonn, Germany.

**Keywords:** case report, features, imaging presentation, Langerhans cell histiocytosis, multisystemic

## Abstract

**Rationale:**

Langerhans cell histiocytosis (LCH) is a kind of rare disease in which dendritic cells proliferate abnormally. It often occurs in children and can involve any tissue and organ. The affected sites usually include bone, skin, pituitary gland, and lungs, while the thyroid gland and external auditory canal are rarely observed. The perineal and labial involvement of this disease has not been reported yet.

**Patient concerns:**

A 47-year-old female patient experienced a swelling of the anterior neck area without an obvious inducement. She noticed a quail egg-like mass on the left side, and the mass increased progressively within 3 months. The anterior neck area was found to be swollen, and some flaky red rashes were seen on the scalp and bilateral external auditory canals.

**Diagnoses:**

Imaging examination showed enlarged thyroid and cervical lymph nodes, multiple low-density nodules in the liver, and reduced signal in the posterior pituitary gland. The biopsy pathological result of the increased left cervical lymph node indicated that LCH was detected.

**Interventions:**

VP regimen (vincristine, dexamethasone per os) and related supportive treatments were given as inducing chemotherapy for 6 weeks.

**Outcomes:**

After the second chemotherapy, the rash on the scalp and external auditory canal improved, and the neck mass was significantly reduced. After the third chemotherapy, the rash was mostly disappeared, while the neck lumps increased during chemotherapy. Thus, clatribine chemotherapy was recommended as the follow-up.

**Lessons:**

Imaging examinations played an important role in the diagnosis and follow-up of the disease, especially ^18^F-FDG PET/CT, which could show multiple involving organs at the same time. When a patient suffering from diabetes insipidus, skin rash, or fever, has a high FDG uptake PET/CT result in multiple tissues and organs throughout the body, it is necessary to consider the possibility of LCH.

## 1. Introduction

Langerhans cell histiocytosis (LCH) is a rare hematologic disorder that occurs (primarily) in children and adults and is predominant in males.^[1]^ While the underlying pathologic cause for LCH is not yet clear, the disease is characterized by an abnormal proliferation of bone marrow-derived histiocytes called Langerhans cells. In addition to pathologic dendritic cells,^[2]^ several immune cells (e.g., T cells, eosinophil, and macrophages) and many cytokines/chemokines have been reported to be involved in LCH lesions.^[3]^ Despite the diverse clinical presentations of LCH, the histologic presentation is primarily consistent with immunohistochemical evaluations for CD1a and Langerin/CD207.^[4,5]^

LCH can involve any tissue and organ. The vulnerable sites are bone, skin, pituitary, lungs, lymph nodes, and liver.^[6]^ But it rarely involves the thyroid and external auditory canal, and there is no report found for perineal and labial infiltration. The case reported in our study described an adult female patient with a rare LCH multisystem infiltration, which had not been previously reported. Imaging examination (e.g., computed tomography [CT], magnetic resonance imaging [MRI], 18F-FDG PET/CT) showed the involved sites were multiple uncommon sites of lymph nodes, liver, and other rare sites (thyroid, external auditory canal, perineum, and labia majora) which were confirmed by pathology. The case report aimed to improve the understanding of imaging examination (especially for 18F-FDG PET/CT) in LCH multisystem infiltration assessment, which might contribute to the diagnosing, staging and treating of patients in time. In addition, we highlighted the advantage of combining 18F-FDG PET/CT together with clinicopathologic, histopathologic, and immunohistochemical markers to identify the extent of systemic invasion of LCH.

## 2. Case description

### 2.1. Chief complaints

Female, 47 years old, with progressive swelling of the anterior neck area for more than 9 months.

History of present illness On June 12, 2020, there was no obvious cause of conscious swelling of the anterior neck area. At first, a quail-egg-like mass was seen on the left side, and the mass increased progressively during 3 months. Other symptoms observed were slight fatigue, but there was no fever, no pain in the anterior neck area, no foreign body sensation, and no general edema. Since suffering the illness, she was sane and mentally weak. On March 6, 2020, the thyroid function of this patient was checked in another hospital. The results showed that the TSH was 9.9 μIU/mL, T3 was 0.58 ng/mL, free T3 and T4 were normal, and the local doctor recommended to increase the dose to 1.5 tablets Qd of levothyroxine sodium for further diagnosis and treatment, on April 23, 2020, she was admitted to the endocrinology department in our hospital with a “goiter.”

### 2.2. History of past illness

The patient had a history of diabetes insipidus for 10 years, regularly took bidantidiuretic hormone (1 tablet bid), and urinated 2 to 3 times a day at night. She had repeated eczema for 10 years and had a history of hypothyroidism. She took Levothyroxine sodium (1 tablet qd) regularly for the past 8 years. Denied diabetes, hepatitis, tuberculosis, contact with water-affected areas, history of smelting, travel, etc.

### 2.3. Family history

There were no other family members showing similar symptoms and nothing of relevance was found in the family genetic medical history.

### 2.4. Physical examination

The body temperature was 37.2°C. Anterior neck area swelled and had a soft texture, and no pain was noticeable when a pressure was applied. There was no pitting edema in the lower limbs, the muscle strength of the limbs was normal, and physiological reflexes existed. Large flaky red rashes could be seen on the scalp and external auditory canal. A new papule-like flaky layer could be seen on the left neck and external auditory canal. The perineum and labia majora were thickened, swollen, and localized, with small patches of erythema.

### 2.5. Laboratory examinations

Free triiodothyronine FT3 3 pmol/L (3.1–6.8 pmol/L), triiodothyronine T3 0.5 ng/L (0.8–2 ng/L), free thyroxine (FT4) 7.2 pmol/L (9–25 pmol/L), thyroid stimulating hormone (TSH) 0.138 μIU/mL (0.27–4.2 μIU/mL), total Vitamin D3 (VITD3-T) 16.88 ng/mL (>20 ng/mL), thyroglobulin, thyroxine (T4), anti-thyroid peroxidase antibody, and TSH receptor antibody were normal. The values of Alpha-fetoprotein, carcinoembryonic antigen, and carbohydrate antigen 199 were in normal range. White blood cell 10.6 × 109/L (3.5–9.5 × 109/L), neutrophil percentage 86.5% (40–75%), lymphocyte percentage (LY%) 9.0% (20–50%), red blood cell 3.34 × 1012/L (3.8–5.1 × 1012/L), hemoglobin 85 g/L (115–150 g/L). There were several results in abnormal status including C-reactive protein 38.5 mg/L (0–8.0 mg/L), lactate dehydrogenase (LDH) 363 IU/L (114–240 IU/L), blood lipids increased, and liver function. In addition, Cortisol (COR) 48 μg/dL (3.5–20 μg/dL), procalcitonin 0.14 ng/mL (0–0.05 ng/mL), ACTH 55 μg/mL (10–52 μg/dL).

### 2.6. Imaging examinations

**Figure 1.**
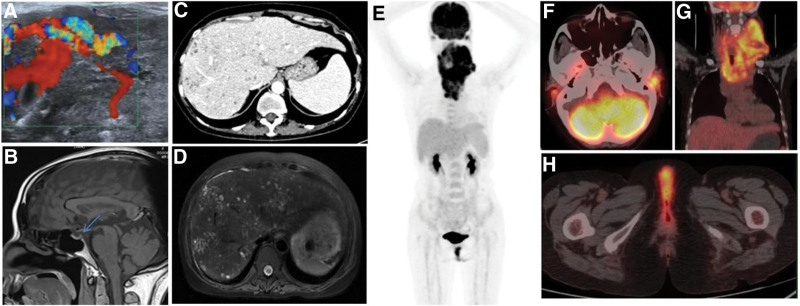
(A) The left thyroid lobe was enlarged and blood flow was abundant as shown by CDFI. (B) In the sagittal T1WI MRI view, the signal from the posterior pituitary lobe was markedly reduced (blue arrow). (C) CT scan showed hepatosplenomegaly with many small round and slightly condensed shadows in the liver. Also increased venous phase with mild enhancement in the marginal area. (D) T2WI view showed multiple small round shadows with high intensity in the liver. (E) The maximum intensity projection of the 18FDG PET/CT whole-body image showed shadows in the bilateral neck, upper mediastinum, and thyroid area. Nodules and streaks with concentrated shadows in the bilateral external auditory canal and perineal area. (F) PET/CT fusion image showed increased FDG uptake of the lesion in the bilateral external auditory canals with a SUVmax of 5.8. (G) PET/CT fusion image showed diffuse thyroid enlargement with increased FDG uptake and a SUVmax of 10.3. Multiple swollen lymph nodes were visible on both sides of the neck. The left side had coalesced into a cluster with increased FDG uptake and an SUVmax of 9.3. (H) PET/CT fusion image in transverse view described increased FDG uptake in the perineum with an SUVmax of 9.9. CT = computed tomography, FDG = fluorodeoxyglucose, MRI = magnetic resonance imaging.

On April 23, 2020, Neck color Doppler ultrasound (Fig. 1A): the thyroid gland was diffusely enlarged, the left lobe was 105 × 81 × 35 mm, the right lobe was 85 × 38 × 32 mm, and the left lobe enlarged significantly, reaching the back of the neck. The internal echo was not uniform, the blood supply was increased, and there was a multiwinding cord-like hyperechoic light band, and bilateral cervical lymph nodes were increased.

On April 25, 2020, head MRI and abdominal MRI standard and enhanced scanning: the posterior pituitary signal was significantly reduced, and the pituitary stalk was not thickened, about 2.67 mm (Fig. 1B). The liver and spleen were enlarged, and extensive small round low-density shadows could be seen in the liver parenchyma, and the edge of the enhanced scanning was slightly enhanced (Fig. 1D).

On April 26, 2020, Neck CT and abdominal CT standard and enhanced scanning (Fig. 1C): the thyroid gland was diffusely enlarged. Multiple lymphadenopathies in the bilateral neck, supraclavicular fossa, left subclavian fossa, and anterior superior mediastinum were big and fused into a mass. The liver and spleen were enlarged, and extensive small round low-density shadows could be seen in the liver parenchyma, and the edge of the enhanced scan was slightly enhanced.

On May 11, 2020, bone marrow aspiration cytology examination observed an infectious bone marrow picture with proliferative anemia (low internal iron).

On May 13, 2020, 18F-FDG PET/CT examination: There was multiple lymphadenopathy on both sides of the neck and anterior superior mediastinum, with a clear fusion on the left side and increasing fluorodeoxyglucose (FDG) uptake. The SUVmax was 9.3 (Fig. 1G). Diffuse thyroid enlargement with increasing FDG intake was detected with the SUVmax of 10.3. The bilateral external auditory canals were thickened which was more prominent on the left side (Fig. 1F). The nodular FDG uptake was increased and the SUVmax was 5.8. The perineum and labia majora strips became thicker. FDG intake increased and SUVmax was 9.9 (Fig. 1H). Small round low-density shadows were frequently seen in the liver. FDG uptake was slightly increased and SUVmax was 2.3. There was an enlarged lymph node near the hepatic portal and retroperitoneum. The FDG intake was slightly increased, and the SUVmax was 2.2. There was no abnormality in pituitary morphology and metabolism.

### 2.7. Further diagnostic checkup

**Figure 2.**
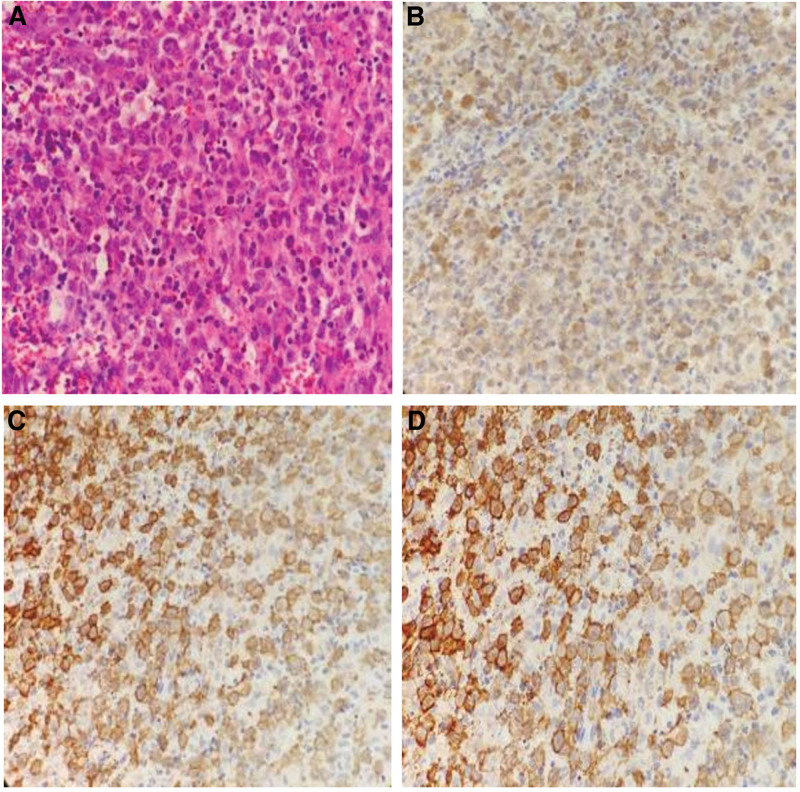
(A) Pathology of the left cervical lymph node, left external auditory canal, and vulvar biopsies by hematoxylin and eosin HE (40×) staining showed that the tumor cells were diffusely distributed and had partial hemorrhage and necrosis (not shown). The tumor cells were large and oval with complex folded and grooved nuclei. The nuclear chromatin is fine, nucleoli are inconspicuous. The cytoplasm is abundant and slightly eosinophilic. The characteristic milieu includes a variable number of eosinophils, histiocytes, and osteoclast-like cells. Immunohistochemical markers are detected as follows: (B) S100 (+), (C) Langerin (+), (D) CDIa (+), CD68 (+ low) CD4 (+) CD31 (+) CD30 (+ partial) Vimentin (+) Ki67 (+ 30%) EBER (−).

The patient underwent biopsy pathology after resection of the left cervical lymph node on April 30, 2020. On May 14, 2020, a needle biopsy was performed on the left external auditory canal and perineum after PET/CT examination. There were medium-sized cells in the cervical lymph nodes, accompanied by massive hemorrhage and necrosis, nuclear mitoses were visible (Fig. 2A). The immunohistochemical markers were observed as following S100 (+) (Fig. 2B), CDIa (+) (Fig. 2C), Langerin (+) (Fig. 2D), CD68 (slightly+), CD4 (+), CD31 (+), CD30 (partly +), Vimentin (+), Ki67 (+30%), EBER (−). The pathological result was suggestive of LCH. The patient suffered from anemia and hypoproteinemia and was transferred to the Department of Hematology on April 30, 2020. 18F-FDG PET/CT examination followed by a biopsy of the bone marrow and perineum in the department of hematology revealed multiple organ involvement throughout the body. The bone marrow cytology indicated infection, and the pathology of the perineum indicated the LCH involvement.

### 2.8. Final diagnosis

The final diagnosis of the presented case is LCH.

### 2.9. Treatment

Vincristine and prednisone regimen, vincristine (once a day, 3 mL each time, intravenously) supplemented with dexamethasone (2 mg, once a week), as the induction chemotherapy lasted 6 weeks and related supportive treatments were given. On May 20, 2020, she received the second chemotherapy. On June 25, 2020, the patient was admitted to the hospital to receive the third cycle of chemotherapy.

### 2.10. Outcome and follow-up

After the second chemotherapy, the rash on the scalp and external auditory canal improved, and the neck mass was significantly reduced. After the third chemotherapy, the rash was mostly disappeared while the neck lumps increased during chemotherapy. Thus, clatrobine chemotherapy was recommended as the follow-up. However, the patient was discharged from the hospital and transferred to another hospital for further treatment.

The complete clinical process of diagnosis and treatment were provided in Table 1.

**Table 1:**
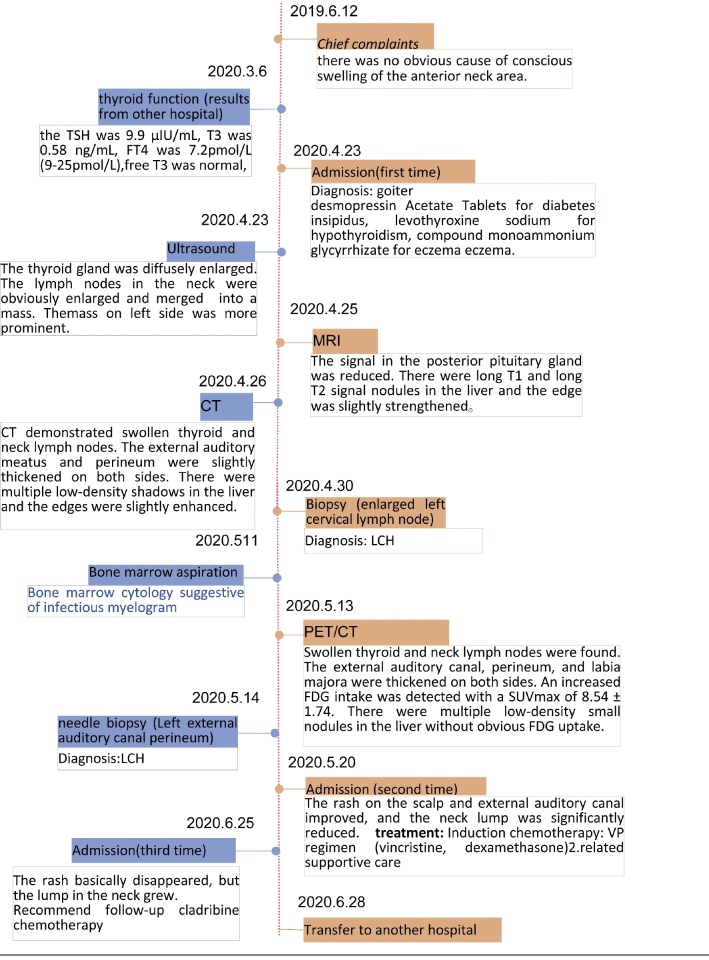
The main timeline of the complete clinical diagnosis and treatment.

## 3. Discussion

It is currently believed that LCH is a clonal hematological tumor characterized by the activation of the mitogen-activated protein kinase signaling pathway. It belongs to the inflammatory myeloid tumors.^[7]^ About 75% of LCH cases have a mutation of mitogen-activated protein kinase pathway activation.^[8,9]^ The clinical, pathological, and imaging manifestations are diverse in LCH, which contribute to the misdiagnosis of LCH. LCH is common in children and the incidence for children under 15 years is 4 to 6 per hundred million per year, but it is seldom happened in adults.^[10]^

Extra axial structures such as the hypothalamus-pituitary gland and dura mater of the nervous system have no blood-brain barrier protection and are the most easily invaded sites for LCH. It has been reported that the initial symptoms of adult patients with hypothalamic pituitary LCH include diabetes insipidus.^[11]^ Central diabetes insipidus is an important clinical manifestation of posterior pituitary involvement and a marker for evaluating LCH disease activity,^[12]^ Enhanced MRI showed a decreased signal of the posterior pituitary gland and/or thickened pituitary stalk (>3 mm). In this case, the signal of the posterior pituitary gland was significantly reduced, but the pituitary stalk was not thickened (about 2.67 mm). LCH lymph node involvement can be part of multiple system infiltration and it can be the initial symptom or even the only manifestation. It usually manifests as swollen lymph nodes while some of which are fused in a lump. Lymph nodes in all regions of the body can be affected. It is more common to occur in the neck with an increased FDG intake. However, it lacks the characteristic imaging appearance and is prone to get false positive results. It is difficult to distinguish between simple lymph node LCH and lymphoma, but PET/CT can guide the biopsy sites. Lymph node LCH can progress to highly malignant Langerhans cell sarcoma.^[13]^ In this case, cervical lymphadenopathy was the first manifestation, which was initially considered as a lymphoma.

Liver LCH is mainly manifested as a diffuse enlargement of the liver, which is caused by the proliferation of Kupffer cells through the massive infiltration of Langerhans cells or the activation of the systemic immune system. It can also appear as a “portal halo sign,” which forms a strip or ring around the portal vein low density shadow. When the histocellular proliferation in the portal area forms focal nodular accumulations, CT shows multiple or diffuse small round low-density shadows in the liver. MRI shows long TI and T2 signals, and the enhanced scanning is slightly enhanced on the edge. The FDG uptake is not obvious. The imaging manifestations of liver infiltration in this case are similar to the above descriptions. LCH rarely invades the thyroid gland, and it is mainly observed as a diffuse or nodular enlargement. Patten et al^[14]^ summarized a total of 75 cases of LCH thyroid involvement reported in the literatures from 1961 to 2012. Total 59% of patients showed a diffuse thyroid ultrasound, 25.8% showed nodular enlargement, and 13.6% showed heterogeneous enlargement, while 1.5% are unknown. Some scholars have pointed out that thyroid LCH can simultaneously present with Hashimoto thyroiditis, thyroid papillary carcinoma, etc.^[15]^ Therefore, for cases where multisystemic LCH is suspected of involving the thyroid, an ultrasound-guided puncture is recommended for the pathological diagnosis. In addition, patients with thyroid involvement need to pay special attention to the presence of liver involvement. Thyroid involvement in this case showed a diffuse thyroid enlargement. The FDG uptake was diffusely increased and the SUVmax was 10.3. LCH external auditory canal infiltration is relatively rare, but infiltration is considered when intractable otitis media with external auditory canal rash occurs repeatedly. However, LCH infiltration of the perineum and labia has not been reported yet. Sometimes vulvar infiltration lesions need to be distinguished from urine contamination. Diuretic imaging or vulvar flushing can help to eliminate the interference of urine. In this case, it was very rare that multiple unusual sites such as liver, thyroid, external auditory canal, perineum, and labia were invaded by LCH at the same time.

The Langerhans cell, histiocytes, lymphocytes, and eosinophils of LCH have high glucose affinity. Therefore, 18F-FDG PET/CT imaging often shows a high glucose metabolism activity when applied in LCH patients. In this case, cervical lymph nodes, thyroid gland, external auditory canal, and perineal lesions all have a significant uptake of FDG with the SUVmax of 8.54 ± 1.74. The enlarged lymph nodes near the hepatic portal and retroperitoneum showing no obvious uptake of FDG may be reactive hyperplasia, but it has not been confirmed by the pathology.

LCH often involves multiple systems and organs of the whole body simultaneously. Traditional imaging (such as CT, MRI, ultrasound, etc) lacks overall features as a local examination and is easy to miss. In addition, traditional imaging methods are difficult to assess the lesion activity accurately. 18F-FDG PET/CT imaging can obtain whole-body images in 1 scan and provide information on the anatomy and metabolic activity of the lesion, which can comprehensively evaluate the involvement of the whole body organs and systems of LCH. Thus, it has many advantages in evaluating multisystemic diseases. Beside the lesion activity, it also can be used for staging, classification, monitoring of recurrence, and finding some hidden lesions. Some studies have shown that 18F-FDG PET/CT significantly improved the accuracy of LCH diagnosis and could evaluate the lesion activity and prognosis.^[16,17]^ PET/CT can accurately distinguish single- and multi-system involvement of LCH, especially the judgment of “dangerous organs” (bone marrow, spleen, liver and lung). It provides a basis for selecting treatment and prognosis evaluation. Practice has proven that PET/CT is a valuable method for evaluating LCH,^[18,19]^ and it has a high sensitivity and specificity to LCH.^[20,21]^ In this study, PET/CT simultaneously detected multisystem involvement of LCH. Auditory canal, perineum, and labia majora infiltration showing a slight thickening of the local tissues in traditional imaging were easy to miss, but could be found by PET/CT due to their significant FDG uptake. Nevertheless, when PET/CT shows negative results due to an absence or low level of FDG uptake as occurs in low-density cystic lesions of the liver and lung, CT or magnetic resonance examination is still an important examination. In addition, 18F-FDG PET/CT lacks sensitivity in the presence of high uptake of 18F-FDG in normal brain tissue. This method is not adequate to evaluate the involvement of the central nervous system LCH in case of nervous system lesions, especially the pituitary. Therefore, enhanced MRI is still an important method for the diagnosis of LCH in the central nervous system.^[22]^ In this article, the hepatic cystic and pituitary lesions were negative on positron emission tomography, but positive on MRI. In addition, lesions with high FDG uptake may cause false positive assessments, which require further histopathological confirmation. For example, the manifestations of lymphoma may be similar to those of cervical lymph node infiltration in this case.

## 4. Conclusion

Our study reported a case of an adult female patient with a rare LCH multisystemic involvement. The imaging manifestation, treatment process, and follow-up data were complete. Imaging examinations played an important role in the diagnosis and follow-up of the disease, especially 18F-FDG PET/CT, which could show multiple involving organs at the same time. Additionally, PET/CT could explore the scope of LCH systemic involvement better and evaluate active lesions. When a patient suffering from diabetes insipidus, skin rash, or fever, has a high FDG uptake PET/CT result in multiple tissues and organs throughout the body, it is necessary to consider the possibility of LCH.

## Author contributions

**Conceptualization:** Amit Sharma.

**Data curation:** Qian Liu.

**Formal analysis:** Ingo G.H. Schmidt-Wolf, Jianlin He.

**Investigation:** Qinyun Zeng.

**Project administration:** Fengxiang Liao.

**Resources:** Huan Hu.

**Writing – original draft:** Xiaofen Li.

**Writing – review & editing:** Yulu Wang.
